# Need for Multidimensional and Multidisciplinary Management of Depressed Preadolescents and Adolescents: A Review of Randomized Controlled Trials on Oral Supplementations (Omega-3, Fish Oil, Vitamin D_3_)

**DOI:** 10.3390/nu15102306

**Published:** 2023-05-15

**Authors:** Carlo Pruneti, Sara Guidotti

**Affiliations:** Clinical Psychology, Clinical Psychophysiology, and Clinical Neuropsychology Laboratory, Department of Medicine and Surgery, University of Parma, 43126 Parma, Italy

**Keywords:** nutrition, diet, oral supplementations, omega-3, fish oil, vitamin D_3_, depression, children, adolescents, mood disorders

## Abstract

(1) *Background*: Depression is a serious health problem with a high cost for public administration. Epidemiological studies report that one in five children have a mental disorder and about 50% of mental health problems exacerbate in childhood and adolescence. Moreover, the antidepressant efficacy in children and adolescents is poorly demonstrated and can cause severe behavioral adverse events such as suicidal ideation. (2) *Methods*: This systematic literature review examined oral supplementations (Omega-3, fish oil, Vitamin D_3_) to treat depressed children, preadolescents, and adolescents. MEDLINE, Scopus, Embase, and PsycInfo were searched for articles published in the last five years. Six studies met the eligibility criteria. The inclusion criteria encompassed children, preadolescents, and adolescents, a diagnosis of depression, and an intervention of oral supplementations such as Omega-3, fish oil, and Vitamin D_3_. (3) *Results*: Most of the studies demonstrated that dietary intervention provides positive outcomes in terms of depression symptoms. (4) *Conclusions*: Overall, the results demonstrate a positive effect for oral supplementation suggesting an increase intake of Omega-3, fish oil, and Vitamin D_3_. However, only a few studies assess the effectiveness of diet recommendations, as a monotherapy or combined treatment, for the management of depression at developmental ages. Thus, there is still a need to further investigate these aspects and to look more specifically at adolescents and preadolescents.

## 1. Introduction

Depression is becoming a serious health problem with a high cost for public administration. As reported by the Global Health Estimates of the World Health Organization (WHO) in 2021 [1], among 10–19-year-olds, one out of seven suffer from a mental disorder globally, which corresponds to 13% of the overall burden of disease. Emotional and behavioral disorders are the most frequent causes of illness and disability among this age group.

Depression is a mood disorder that affects the emotional, cognitive, and behavioral dimensions and negatively affects the whole functioning of the person [2]. Not taking care of adolescents’ mental health conditions can have a long-term impact, impairing both physical and mental health and compromising the ability to live a fulfilling life as an adult. Therefore, depression is a frequent disorder but is often underestimated due to the social stigma [3,4].

Epidemiological studies estimate that one out of five children have a mental disorder before reaching adulthood [1,2,5]. Depression has been ranked as the second leading cause of death in adolescents due to suicidal behavior [5,6]. Furthermore, assuming that 50% of adult health problems arise in childhood and adolescence [7], the need to pay attention to this age strongly emerges.

Since the introduction of selective serotonin reuptake inhibitors (SSRIs), including fluoxetine, sertraline, paroxetine, fluvoxamine, citalopram, and escitalopram, promising results for the pharmacological treatment of major depression, obsessive-compulsive disorder, generalized anxiety disorder, panic disorder, and social anxiety disorder were found [8]. Due to the perceived safety of these drugs and their convenience (e.g., single-day dosing, no need to monitor blood levels), SSRIs have dominated the market. Although most research has focused on adult populations, large-scale studies were also conducted in pediatric settings [8,9,10]. Thus, a sharp increase in the prescription of these psychotropic drugs, even in childhood, was observed [9,11]. However, the Food and Drug Administration (FDA) issued an antidepressant warning for the risk of suicidal thoughts and behaviors in children and adolescents based on the results of clinical trials conducted at the turn of the new millennium [11,12]. In this regard, there were research findings supporting that venlafaxine posed a significantly increased risk of suicidality (suicidal ideation or even attempts) for young people. This dangerous side effect was associated with a particular antidepressant-related adverse event—activation, which is a hyper-arousal event that is typically characterized by specific symptoms including an increase in activity, impulsivity, disinhibition, restlessness, and insomnia [13,14]. These data led mental health professionals to drastically reduce antidepressant prescribing [15,16,17].

An alternative to psychiatric drugs seems to derive from nutritional neuroscience [18,19,20], an emerging field of research that explores nutritional factors related to human cognition, behavior, and emotions. During the past fifteen years, a good amount of research has suggested that diet could play an important role in the treatment and prevention of depression. The research examining this relationship belongs to two different approaches, which investigate either the impact of individual nutrients such as n-3 fatty acids [21] or vitamins and minerals [22]. Additionally, some intervention studies examined the effect of supplements (i.e., multivitamins, eicosapentaenoic acid (EPA), and docosahexaenoic acid (DHA)) on mood [23]. Other studies evaluated the relationship between diets as a whole and their effects on mental health [24,25,26]. The research looking at these different approaches uniformly concluded that the relationship is potentially bidirectional and therefore complex [27]. Nevertheless, the relationship between nutritional status and mental health in young people and children deserves specific attention since teens generally become more independent and make more decisions about the type and amount of food they consume, including “junk” and “fast” foods.

With this in mind, diet modification might be considered as a goal for the prevention of mental disorders. At the same time, nutritional adjustment should be evaluated as a fundamental part of the integrated intervention in which the psychiatric treatment is complemented by mental health research, education, policy, and promotion [4,20,28]. Recent reviews [24,25,26] agree that daily supplementation of specific nutrients is often effective in reducing depressive symptoms in adults. In particular, the researchers’ perspectives are based on the fact that specific amino acid supplements favor the production of neurotransmitters involved in brain functioning and alleviate the symptoms of mental disorders, including depression (Table 1). For instance, surprising findings were described by Ano and colleagues [29], who demonstrated through animal models that specific supplements or nutraceutical compounds in whey digest rich in β-lactolin are able to increase dopamine levels in the area of the frontal cortex associated with depressive manifestations.

In light of the current literature, the aim of the present review was to investigate the efficacy of food supplements in reducing depressive symptoms in children, preadolescents, and adolescents. Only randomized controlled trials (RCTs) were included in order to better define the causal role of nutrition (healthy diet and food supplements) on mood.

## 2. Materials and Methods

### 2.1. Literature Search

Taking into account that an in-depth literature review was recently carried out by some colleagues [6], MEDLINE, Scopus, Embase, and PsycInfo were searched for articles published in a very recent period—the last five years. The following search terms were used: Depression AND (Children OR Adolescents OR Teenagers) AND (Diet OR Food OR Nutrition OR Vitamins OR Minerals OR Probiotics). Two reviewers independently evaluated publications for inclusion, based on their titles and abstracts. Full texts were then retrieved for those articles deemed eligible and considered for inclusion independently by both reviewers.

### 2.2. Inclusion and Exclusion Criteria

Studies were included in the review if they: (1) examined a sample of children, adolescents, and preadolescents (≤19 years of age); (2) quantitatively assessed the depressive symptomatology through a recognized scale (i.e., Children’s Depression Rating Scale (CDRS) [31], Beck Depression Inventory (BDI) [32], or Children Depression Inventory (CDI) [33]); (3) were RCTs; (4) were published in peer-reviewed journals; and (5) investigated the effectiveness of micronutrients in reducing the depressive symptoms. Studies were excluded if they: (1) were qualitative, quasi-qualitative, reviews, meta-analyses, case studies, theses, dissertations, or conference presentations; (2) did not specifically measure depression and depressive symptoms; and (3) enrolled adult patients.

### 2.3. Quality Appraisal

The Joanne Briggs Institute (JBI) Critical Appraisal Checklist for Randomized Controlled Trials was selected to assess the quality of records [34]. Two reviewers independently assessed the quality of studies and resolved discrepancies by discussion.

### 2.4. Data Extraction

Data relevant for the review included details about participants, the assessment of depression, and the evaluation of the effectiveness of micronutrients in reducing the depressive symptoms. Additional extracted data included year of publication, sample size, study design (i.e., RCT), and data analysis (e.g., Independent Sample T Test or Mann–Whitney U Test).

## 3. Results

### 3.1. Study Selection

**Figure 1:**
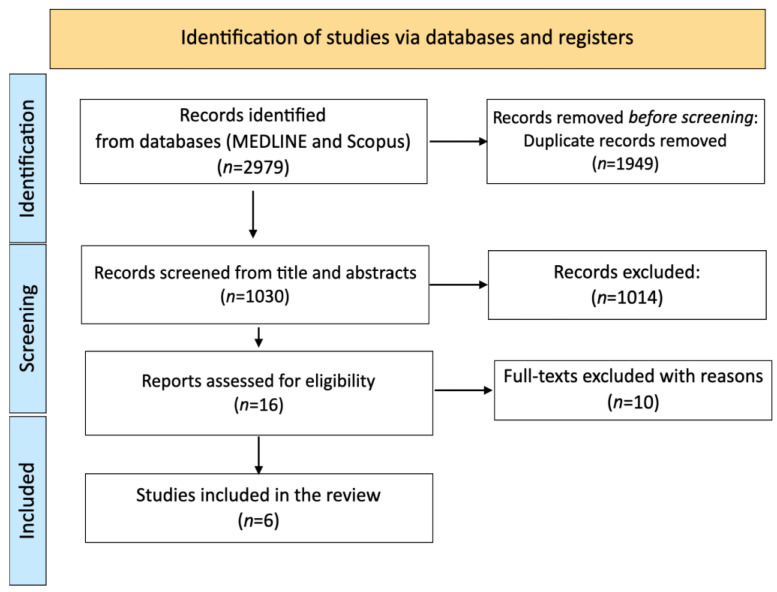
PRISMA flow diagram showing the selection of primary studies.

The initial search resulted in 2979 studies. Of these, 2965 were eliminated through the initial screening process. Of the remaining 16 studies, 10 were not considered due to incorrect intervention (*n* = 2), protocol paper (*n* = 2), incorrect study design (e.g., cohort study, *n* = 2), clinical trial (*n* = 1), different outcomes assessed (*n* = 2), or duplicated data (*n* = 1). Six studies were included in the review. In Figure 1 the outcomes of the study selection process are presented.

### 3.2. Study Characteristics

#### 3.2.1. Population Characteristics

All studies targeted young populations (e.g., ≤19 years), except one [35] that included participants aged from 9 to 21 years. Considering the symptomatology, five studies identified existing Major Depressive Disorder (MDD), Mixed Anxiety and Depressive Disorders (MADD), Dysthymic Disorder (DD), or Depressive Disorder Not Otherwise Specified (DD-NOS) [35,36,37,38,39]. Among this research, one study required that the participants had at least one biological parent with Bipolar I disorder [35]. One study targeted adolescents that had clinically significant symptoms as reported by the BDI (total score > 13 points) as well as D hypovitaminosis [40].

#### 3.2.2. Intervention Style

Three studies utilized a supplementation of fish oil for the experimental group and had a placebo group [35,37,38]. Three studies compared the effectiveness of a combined treatment. Among these three, two studies provided an oral supplementation of Omega-3 with Psycho-Educational Psychotherapy (PEP) to the experimental group [36,37], while the third study provided an oral supplementation of Vitamin D_3_ with Treatment As Usual (TAU) (in agreement with the clinical guidelines) for the experimental group [40]. Although the term “probiotics” was among the search keywords, no results were found.

A Local Institutional Review Board approved all the studies, and informed consents were obtained from all of the participants of the studies.

#### 3.2.3. Quality Assessment of Included Studies

The results of quality assessment using the JBI critical appraisal checklist for RCTs are summarized in Table 2. Overall, the quality of the studies was medium-high.

### 3.3. Results for Depression

A summary of the characteristics of the analyzed studies is shown in Table 3.

The study by McNamara et al. [35] involved preadolescents (aged 9 to 11 years) who met the criteria for a depressive disorder according to DSM-IV [41] or who reported significant depressive symptoms detected by the Children’s Depression Rating Scale-Revised (CDRS-R) [42,43]. Furthermore, a requirement for participation in the research was a family history of mood disorders (at least one parent with Bipolar I disorder). Of the 56 patients involved, 42 completed the 12-week study. Moreover, 21 subjects represented the control group and another 21 followed the experimental treatment with fish oil intake (2100 mg/day). The primary outcome measure was the change in the total score of the CRDS-R. The secondary outcome measures used were the Young Mania Rating Scale (YMRS) [44] as well as the Clinical Global Impression-Severity (CGI-S) and Clinical Global Impression-Improvement (CGI-I) from the Children’s Global Assessment Scale (CGAS) [45,46] and Child Behavior Check-List (CBCL) [47]. The two treatment groups (*p* = 0.15) did not differ in CDRS-R scores, but a significantly greater decrease in CGI-S (*p* = 0.0042) and CGI-I (*p* = 0.036) scores emerged in the experimental group in comparison with the placebo one.

Another study looking at the efficacy of Omega-3 monotherapy is that of Trebaticka et al. [37]. These investigators involved 60 outpatients (aged 7 to 18 years) with a diagnosis of depressive disorder (*n* = 11) or MADD (*n* = 29). The experimental group received an Omega-3 fatty acid-rich fish oil emulsion (Om3 group) while the control group took an active Omega-6-rich sunflower oil emulsion comparator (Om6 group). Both groups underwent treatment for 12 weeks, followed by a 4-week washout period. The CDI [33,48] was administered at baseline and every 2 weeks throughout the intervention period. Researchers concluded that a fish oil emulsion rich in Omega-3 fatty acids might be an effective adjuvant supplement when treating depressive disorders in young patients. A significant reduction in CDI scores was observed after 6 and 12 weeks of the intervention. In particular, the time-dependent treatment effect in the Om3 group was significant (F = 6.284, *df* = 6, *p* < 0.0001) compared to the Om6 group (F = 1.36, *df* = 6, *p* = 0.233). In the Om3 group, analyses showed significant differences in CDI scores from baseline (*p* = 0.001 in week 2 to *p* < 0.0001 in week 12). The highest reduction in CDI score in the Om3 group was recorded after 10 weeks of intervention (−7.6, −27.4% of the baseline score). However, the effect of treatment in time was significant in Om3 group with DD (*p* < 0.0001), unlike the MADD group (*p* = 0.201).

Unfortunately, Gabbay et al. [38] did not obtain similar results. These researchers involved a group of teenagers (48 completed the study) diagnosed with MDD and randomized them between the experimental and control groups. The subjects belonging to the first group took Omega-3 fatty acids in the following way: each participant started with an initial dose of 1.2 g/day, then the doses were increased in increments of 0.6 g/day every 2 weeks (maximum possible dose of 3.6 g/day, combined EPA [2.4 g] + DHA) [1.2 g])). Contrary to other studies conducted, taking the supplement did not demonstrate superior efficacy to the placebo in reducing the severity of symptoms of depression, which was investigated through the CDRS-R [43] or the BDI-II [32].

Over the past five years, Fristad and colleagues [36,39] have conducted an RCT and a follow-up study with the intent of decreasing the symptoms of depression in young patients. In 2019, Fristad et al. [36] psychologically screened a large group of preadolescents and adolescents (178 persons between the ages of 7 and 14). The randomized study involved 37 patients with MDD; 5 with DD; and 30 with DD-NOS. In the intervention, which lasted 12 weeks, one group took an Omega-3-based supplement (two capsules of 500 mg Omega-3 each (350 mg EPA: 50 mg DHA, a 7: 1 ratio; 68 mg more Omega-3) twice a day, for a total daily dose of 1870 mg Omega-3). Another group underwent PEP, a family-based Cognitive Behavioral Therapy (CBT), and took a placebo. A third group combined the Omega-3 together with PEP intervention. Finally, a last group only took a placebo. The placebo groups received two placebo capsules twice daily, matched to Omega-3 for smell and appearance. The severity of depressive symptoms was assessed at baseline and at 2, 4, 6, 9, and 12 weeks through the CDRS-R [43]. The highest percentage of depressive symptoms’ remission was recorded for the group treated with combined intervention (Omega-3 + PEP = 77%) and by the treatment group with PEP and placebo (61%); 44% of symptoms’ remission was detected for the Omega-3 group; however, a benefit also emerged within the placebo group (56%). In addition, intent-to-treat analyses found low-medium effects for the combined treatment (d = 0.29) and Omega-3 alone (d = 0.42). Within a follow-up study [39], the same authors evaluated the outcomes on symptoms of depression (*n* = 25) or sub-syndromic Bipolar disorder (*n* = 13), reassessing young people involved in the RCT 2–5 years earlier. The intervention included the intake of Omega-3 fatty acids (Omega-3), Individual Family PEP (IF-PEP), and a combined treatment. Overall, from baseline to follow-up, mood severity (CDRS-R [43]: t = 2.13, *p* = 0.04, d = −0.035), executive functioning (Behavior Rating Inventory of Executive Functioning (BRIEF) [49]: t = −4.07, *p* = 0.000, d = 0.60), and the global one (CGAS [45,46]: t = 3.89, *p* = 0.000, d = 0.63) continued to be significantly improved even if depressive symptoms (CDRS-R [43]: t = 7.69, *p* = 0.002, d = −0.71) increased significantly from the end of the RCT study to the follow-up.

Finally, Libuda et al. [40] focused their attention on the association between Vitamin D_3_ deficiency and mood. These authors involved patients with confirmed Vitamin D hypovitaminosis [25 (OH) D ≤ 30 nmol/L] and with at least mild depression, assessed through the BDI-II [32] (score > 13). First, a Vitamin D deficiency was diagnosed in 138 (49.3%) of the 280 screened patients. In addition, 113 (40.4%) participants met all inclusion criteria and were randomized in a double-blind manner into the experimental group (receiving 2640 IU, or 66 mg/day, of Vitamin D_3_/d) or placebo. Additionally, in this study, it was a question of verifying the efficacy of the food supplement in addition to psychological intervention. In fact, both groups simultaneously followed the TAU (in accordance with the clinical guidelines). The BDI-II scores were assessed as a primary outcome, while the scale Diagnostic System for Mental Disorders in Childhood and Adolescence, Self and Parental Assessment (DISYPS-II) [50] and the serum total 25 (OH) D were considered as secondary outcomes. Although the intervention resulted in a greater increase in 25 (OH) D levels in the experimental group than in the control group (treatment difference:+14 ng/mL; 95% CI 4.86–23.77; *p* = 0.003), BDI-II scores did not differ significantly (+1.3; 95% CI −2.22 to 4.81; *p* = 0.466). Nevertheless, significant differences emerged between the assessments made with the DISYPS-II scale by the parents of the patients belonging to the two groups (−0.68; 95% CI −1.23 to −0.13; *p* = 0.016).

## 4. Discussion

This review of the literature analyzed studies examining the relationship between specific nutrient intake and depressive symptoms in children, preadolescents, and adolescents. Since it is important to define the causal role of specific oral supplements on mood and behavior, only interventional RCT studies were considered. Three out of the six studies analyzed and evaluated the effectiveness of oral fish oil (Omega-3) supplements in improving symptoms in preadolescents and adolescents diagnosed with depression. Two out of these three studies demonstrated that Omega-3-based dietary supplements were effective in improving symptom severity [35,37], despite a placebo effect being described by Fristad et al. [36,39]. Effectively, among the clinical trials for depression, the placebo response rate was remarkably high, reaching 30–40% of the patients [51]. Trebaticka et al. [37] found that people suffering from MDD received a greater benefit from this supplementation in comparison with patients diagnosed with MADD. In accordance with the authors, this result highlighted the need to investigate the psychophysiological level and differentiate clinical conditions characterized by anxious activation (typical of the hyper-activation observed in anxious disorders and stress-related syndromes) or psychophysical exhaustion (the autonomic hypo-activation typical of depression). Evidently, this caution could have repercussions on a therapeutic level of considerable importance [52,53]. Two other studies [36,39] administered to depressed preadolescents and adolescents an integrated treatment by adding the Omega-3 supplement to a psychological intervention. In particular, Fristad et al. [36] noted significant improvements in subjects undergoing oral Omega-3 supplementation in addition to PEP. Similarly, in a subsequent follow-up study, the same authors [39] confirmed these results after 2–5 years, even if after the suspension of the supplement a worsening of depressive symptoms emerged. These findings underlined the need to pay constant attention to the nutrition of young persons and to integrate any deficiencies with oral supplements. Another study [40] focused on the dietary supplementation of Vitamin D_3_ in adolescents with confirmed hypovitaminosis and depression. These researchers subjected the young people to integrated treatment (psychological intervention in agreement with the national guidelines) along with oral Vitamin D_3_ supplementation. In this research, no significant differences emerged between the BDI-II scores in the two groups. Instead, an interesting finding is a significant improvement in the symptoms reported by the parents. As sometimes happens in clinical research [54], these data probably highlighted a different trend between the remission of behavioral symptoms (noted by parents) and cognitive-emotional ones (reported by young patients). Therefore, these results certainly need to be replicated and deepened because the possibility of stemming the behavioral symptoms of adolescents (which can sometimes be as serious as suicide) appears to be a health priority [5,8,9,10,17].

Although the data in this review derive from studies conducted on a few patients, their findings offer important insights. First of all, it is necessary to underline that these results regarding the juvenile population are valuable for at least three main reasons: (1) the depressive syndrome in young people is difficult to isolate and differentiate from anxious hyper-activation and somatization that are frequently associated; (2) it is not always easy to obtain a good level of compliance in this category of patients; and (3) not all families of minors willingly accept treatment (even if not pharmacological). However, relevant clinical implications derive from this review and confirm previous works in this regard [4,6,9,20]. Additional data confirming the hypothesis that a correct nutritional style can support the psychophysical balance of young people were provided. In particular, highlighting that specific nutrients can be included in the diet of children, pre-adolescents, and adolescents offers valuable support to their diet. In other words, young people could be educated on a healthy and balanced diet for their physical and mental health. Furthermore, specific food supplements could be able to replace psychotropic drugs for the management of mood alterations, avoiding the risk of life-threatening side effects for patients. Currently, the elective treatment of depression is still psychological therapy [9,36,39,55,56], and the available antidepressants do not guarantee a remission of symptoms [15,16,17]. Indeed, the antidepressants most frequently prescribed to young people (tricyclics such as Imipramine and serotoninergics such as Paroxetine) can further aggravate the patient’s suffering, increase the likelihood of medication discontinuation, and potentiate symptomatic impairment as well as the onset of self-harming behaviors [9,17,19,57]. These data suggest that there is a great need to identify alternative or additional therapies to those currently available. Moreover, it is still necessary to identify useful molecules to prevent the onset of behavioral adverse events as serious as self-injurious gestures. Any strategy aimed at preventing the development of depressive disorders or reducing psychopathological symptoms has large potential as a public health intervention [1,2].

**Figure 2:**
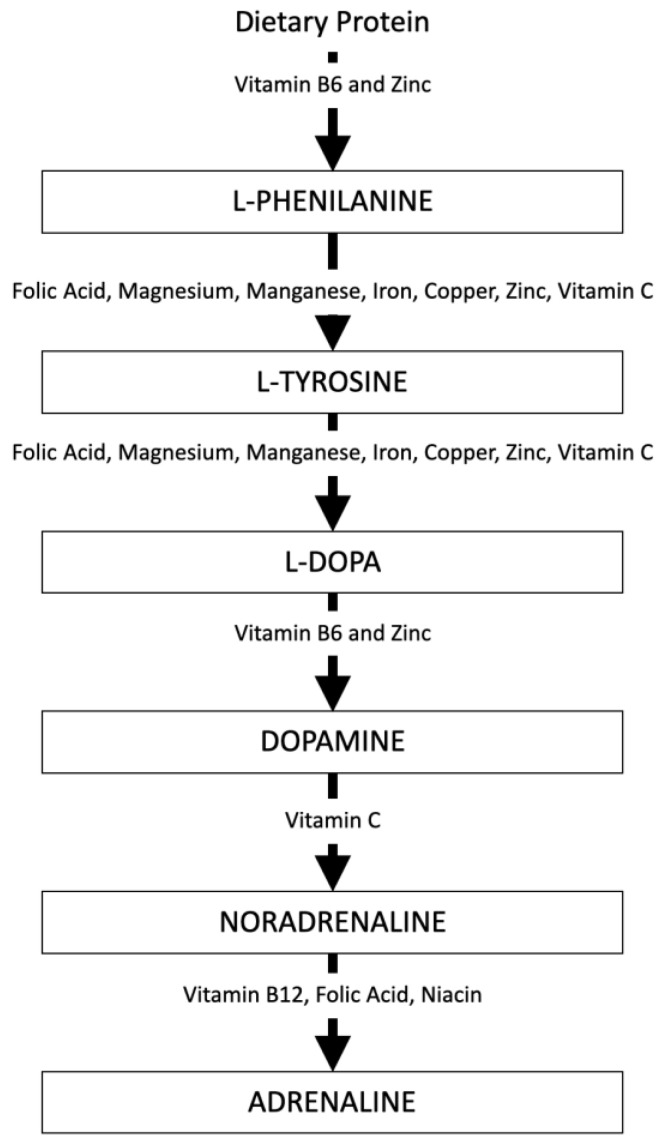
Nutrients and metabolites intake necessary for the synthesis of neurotransmitters. Source: Adapted from Holford [30].

However, it is important to underline that the study of the effectiveness of individual nutrients on mood enhancement is so complex because several neurochemical pathways influence mood regulation. For instance, the release of individual neurotransmitters including serotonin, dopamine, noradrenaline, and adrenaline requires different nutrient and metabolite intakes [4,20] (Figure 2).

In general, researchers’ objective is to investigate the effects of one or more elements involved in the production chain of those neurotransmitters responsible for depression. More specifically, the most widespread biochemical theory identified among the causes of depression is an imbalance in the level of serotonin, which mainly affects mood, and catecholamines (dopamine, noradrenaline, and adrenaline), which also involve motivational aspects (apathy, asthenia, abulia, and anhedonia are the effects of their deficiency) [30]. When considering serotonin, it is important to remember that its precursor is the amino acid tryptophan, a protein constituent. In adults, the recommended dosage of this amino acid is 100 mg of 5 hydroxy tryptophan (5-HTP), two–three times per day, for depression. Some supplements also add vitamins and minerals such as B12 and Folic Acid, which help convert 5-HTP to serotonin [4,20].

As to the catecholamines (dopamine, noradrenaline, and adrenaline), it should be remembered that they are all synthesized starting from the amino acid phenylalanine, which, together with L-Tyrosine, leads to dopamine. Thereafter, Vitamin C, Vitamin B12, Folic Acid, and Niacin allow the synthesis of norepinephrine and adrenaline [4,20]. Recent research on adults states that the best results can be obtained by integrating all of these 5-HTP amino acids, phenylalanine and L-Tyrosine, B vitamins (B6, B12), and Folic Acid [20,30].

Furthermore, Omega-3 fatty acids can directly affect the production and reception of the serotonin. To summarize, the brain gray matter, which at 50% contains polyunsaturated fatty acids (PUFAs) (33% being Omega-3 fatty acids), needs these substances to be introduced in the diet [30]. More specifically, n-3 PUFAs modulate the communication mechanism of brain neurons (DHA makes up 15% of the brain and the EPA constitutes 0.2%). Thus, the DHA concentration influences the permeability of the neural cell membrane, and its deficiency is linked to a reduced and/or dysfunctional transmission of neurotransmitters [20]. Consistent with what was demonstrated by the studies conducted on preadolescents and adolescents and analyzed in the actual review, the consumption of fish (rich in n-3 PUFAs) would be a valuable aid for the treatment of alterations of the central nervous system such as in the case of psychiatric diseases [35,36,37]. The scientific evidence suggests that the depletion of n-3 PUFAs may be an etiological variable of depression as if the reduced availability of production of this neurotransmitter were an important contributor [58,59,60]. Nonetheless, Omega-3 PUFAs have potential benefits for human health as they exert different effects also on the gut immune tolerance and gut microbiota maintenance. More specifically, the important roles of Omega-3 PUFAs lie in their ability to maintain the balance between gut immunity and gut microbiota [61]. Furthermore, accumulating evidence from animal model studies indicates that the interaction between the gut microbiota, Omega-3 fatty acids, and immunity helps maintain intestinal wall integrity and interactions with host immune cells. Consequently, human studies have confirmed the ability of Omega-3 PUFAs to influence the gut–brain axis, acting through the composition of the intestinal microbiota [62]. In agreement with some researchers [63], this aspect would be crucial within major depression, which would be closely related to the health condition of the brain–intestine axis and the conditions of the intestinal microbiota, or even be a direct consequence [63]. Additionally, fish intake (>150 g/week) is associated with a reduction in levels of pro-inflammatory markers including C reactive protein (CRP) and cytokines such as interleukin-6, highlighting the probable further involvement of other processes, including the inflammatory ones [64]. In addition, these mechanisms are already strongly debilitated in conditions of psychological stress [65,66,67]. For instance, a large area of research is currently taking into account the inflammatory factors associated with various mechanisms including the oxidative stress [68]. It is now widely believed among researchers that people with depression have higher levels of oxidative stress and a decrease in antioxidant defenses [69].

In light of these findings, it is comprehensible why a review by Holford [30] concluded by listing a series of specific recommendations for adults useful for making a diet healthy. In addition to exercise and conducting outdoor activities, some oral supplements that may help adults’ psychophysical health include the following: (1) reduction of sugar and stimulants (caffeinated drinks and smoking); (2) augmentation of fruit and vegetables (five portions a day); (3) fatty fish (mackerel, tuna, salmon, herring) intake at least two times a week; and (4) ensuring a sufficient amount of protein from fish, meat, eggs, beans, and lentils. Suggested supplements would be: (1) B complex, including Vitamin B6 (10 mg), Folic Acid (400 mg), and Vitamin B12 (10 mg); (2) additional bursts, 400 to 2000 mg per day; (3) 5-HTP (200 ± 300 mg per day); and (4) Omega-3 rich fish oil, two capsules per day, for at least 400 mg of EPA.

In light of the knowledge that we have with regard to the biological mechanisms, it is important to keep in mind the complexity of the different variables that are involved in the physiopathogenesis of depression and its behavioral manifestations. In agreement with the biological theories, specific brain structures and the function of some neurotransmitters effectively play a pivotal role. Important studies focused on the specific polymorphism of the serotonin transporter in the promoter region (5-HTTLPR) and on the binding site of transcription factors, supported a body of research looking at the association between the short allele (S) for the serotonin transporter and reduced transcriptional activity [70,71]. Notwithstanding, the biological aspect is not the only one to pay attention to. The most innovative finding was that the experiences lived by the person, in relation to their emotional load, constituted a risk factor for the exacerbation of this biological condition. In other words, the genetic predisposition represents the biological substrate on which depression develops. More specifically, Caspi et al. [72,73] found that individuals with one–two copies of the serotonin transporter S allele had a higher probability of being diagnosed with MDD following stressful life events [74]. Other researchers linked this association between genes and environment to a sort of endocrine dysfunction detectable at the level of the Hypothalamic–Pituitary–Adrenal (HPA) axis. The HPA axis would be more sensitive and easily activated if the person experienced stressful or traumatic events during the developmental period [75,76,77]. On a psychological level, this characteristic corresponds to the presence of personality traits including Harm Avoidance, which predispose the person to a greater reactivity to stress both through the involvement of serotoninergic activity and through the hyperactivity of the HPA axis [78].

Thus, the biological dimension could represent the beginning of a path that encounters personality traits that, in turn, make the subject more vulnerable to stressors (i.e., demands deriving from the environment or from the organism itself including biological alterations). Alternatively, behavioral modifications (i.e., withdrawal and isolation typical of depressive states) could generate changes in diet and lifestyle by interfering with the intake of micronutrients involved in the management of psychophysical health. Consequently, given the strong influence of the biological basis of depression, especially at a developmental age, it is so important to pay attention to the psychosomatic and autonomic symptoms. Until the advent of the DSM-III [79], the psychiatric approach did not allow a differentiation of depressive syndromes depending on the age of the individual because it was thought that the clinical condition of children and adolescents was similar to that of the adults [80]. On the other hand, in DSM-IV [41] and DSM-5 [81], some symptoms are considered characteristic of depressive manifestations in childhood and/or adolescence. In addition, some clinical indicators allow the clinician to better identify the depressive disorder depending on the patient’s age. In particular, irritable mood and sadness would be more frequent in children while psychosomatic reactions would be more frequent in adolescents. In general, the behavioral symptoms of developmental depression are various (i.e., hyperactivity, irritability, aggression, etc.) and associated with different psychosomatic manifestations (e.g., headache, abdominal pain, sweating, etc.) [82,83,84]. Typically, the symptoms can be grouped into four areas: (1) Psychomotor slowing, reduced ability to concentrate and think; (2) Psychosomatic reactions such as headaches, sleep disturbances, eating irregularities, etc.; (3) Sadness and disinterest in usual activities, including recreational activities; and (4) Feelings of distrust, low self-esteem, and learned helplessness. These are the symptoms that most affect school functioning, which, in turn, are also influenced by academic performance [85,86].

In some cases, autonomic hyper-activation can favor the appearance of behavioral symptoms very different from the “classic” clinical condition of depression. The latest version of the DSM [81] makes it possible to diagnose Disruptive Mood Dysregulation Disorder (DMDD) for children up to 12 years of age. The main feature of DMDD is chronic, severe, and persistent irritability manifesting as persistent or chronically angry mood and frequent outbursts of anger. Furthermore, children with this disorder are more prone to developing a form of depressive disorder or anxiety disorder (AD) as they enter adolescence or adulthood [82,83]. Moreover, depressive episodes are frequently associated with disruptive behaviors, attention deficit disorders, anxiety disorders, substance abuse, and eating disorders in children, but even more so in adolescents [27,28].

In addition, it is difficult to treat and identify depression at a developmental age, especially because of the comorbidities linked to autonomic dysfunction and its associated somatic complaints. In these cases, the risk of misdiagnosis is high and neglecting a condition of psychophysiological hyper-activation can cause iatrogenic damage. For instance, prescribing antidepressants in a depressive exhaustion syndrome can cause a hypomanic-like reaction two or three weeks after the first intake [87,88]. Sometimes, manic mood imbalance can be interpreted as a drug-induced mania disorder that can lead to life-long prescription of mood stabilizers [53,54,89]. 

In light of the complexity of depressive disorders in childhood and adolescence, research over the past two decades has produced many studies regarding the efficacy of integrated pharmacological and psychological interventions [55,56,90,91,92,93]. Currently, psychotherapies, especially CBT and interpersonal psychotherapy, appear to be more effective than controls as a first-line treatment [55,92,94]. Recent meta-analyses [15,16] highlighted that antidepressants, with the exception of fluoxetine, did not offer a clear advantage over the placebo pill for many individuals. The average effects of antidepressants for MDD in comparison with the placebo pill are not as beneficial as those found for the treatment of other youth problems, including AD and Obsessive-Compulsive Disorder (OCD) [94,95,96]. Additionally, in a recent meta-analysis by Zhou et al. [9], the authors found that the best intervention for children and adolescents with depressive disorders was the integrated treatment (fluoxetine plus CBT).

Although the studies conducted on adolescents are few, the scientific evidence agrees that there is the need to provide a multidimensional assessment of the distress experienced by young people in order to offer the best treatment possible. Carrying out comprehensive “patient care” allows us to satisfy the ethical principles implied by the term “therapy”. The etymology of “therapeia” refers to the concept of “holding” and “supporting”, and this, especially today in light of the discomfort caused by the pandemic, is a clinical imperative [97].

## 5. Conclusions

Notwithstanding, the involvement of different nutrients supports the need to validate the effectiveness of diets, in their complexity, for improving mood and mental disorders. However, despite the results on adults being promising, there is still a need to further investigate these aspects and to look more specifically at adolescents and preadolescents.

To conclude, the interaction between different factors is complex. Genetic predisposition and neurochemical, psychophysiological and psychobiological mechanisms intervene in equal measure. On a psychological level, stressful life events can exacerbate risky conditions and stress can overload the autonomic nervous system. All of these considerations support the need to take a multidimensional and multidisciplinary approach to study, focusing on a complex disease such as depression to intervene on a preventive and therapeutic level. Future studies are needed to validate the effectiveness of combined therapies in preventing and/or treating altered mood at a developmental age or reducing antidepressant-related adverse events.

## Figures and Tables

**Table 1:** Amino acid precursors of neurotransmitters and effects of their deficiency on mental health.

Neurotransmitter	Precursor	Effects of Deficiency	Foods to Consume
Acetylcholine	Choline	Deterioration of memory and imagination; Fewer dreams; Increased confusion, forgetfulness, and disorganisation	Organic/free-range eggs; Organic or wild fish—especially salmon, mackerel, sardines, and fresh tuna
Serotonin	Tryptophan	Low mood, depression; Disrupted sleep cycle; Anxiety	Fish; Lean, organic poultry; Fruits, Avocado; Eggs; Low-fat cheese; Wheatgerm
Dopamine	Phenylalanine	Lack of focus and motivation; Poor attention and memory	Regular, balanced meals; Fruits and vegetables high in Vitamin C; Foods rich in B_6_ and Zinc; Wheatgerm; Fermented products
Norepinephrine and Epinephrine	Tyrosine	Low mood; Anxiety; Poor focus	Foods rich in Folic Acid; Magnesium, Manganese, Iron, Copper, Zinc, Vitamin C
Gamma-Amino Butyric Acid (GABA)	Glutamine	Self-criticism; Anxiety; Unable to relax	Dark green vegetables; Seeds and nuts; Potatoes; Bananas; Eggs

Source: Adapted from Holford et al. [30].

**Table 2:** Critical appraisal tool according to Joanne Briggs Institute checklists.

	Q1	Q2	Q3	Q4	Q5	Q6	Q7	Q8	Q9	Q10
McNamara et al. [35]	Y	Y	Y	Y	Y	Y	U	U	Y	Y
Trebaticka et al. [37]	Y	Y	Y	Y	Y	Y	U	N	Y	Y
Gabbay et al. [38]	Y	Y	Y	Y	Y	Y	Y	N	Y	Y
Fristad et al. [36,39]	Y	Y	Y	U	U	U	Y	Y	Y	Y
Libuda et al. [40]	Y	Y	Y	Y	Y	Y	Y	Y	Y	Y

Note: Y = yes; N = no; U = unclear.

**Table 3:** Characteristics of Included Studies.

Authors	Sample	Intervention	Measures	Outcomes
McNamara et al. [35]	*n* = 56 (9–21 years old). Inclusion criteria: (1) diagnosis of MDD or DD-NOS; (2) a CDRS-R score ≥ 40; (3) at least one biological parent with Bipolar I disorder.	Group 1: fish oil (2100 mg/day); Group 2: placebo; Duration: 12 weeks.	Primary outcome measure: CDRS-R; Secondary outcomes measures: YMRS, CGI-S, CGI-I, CGAS, CBCL	A significant decrease in CGI-S (*p* = 0.0042) and CGI-I (*p* = 0.036) scores was greater for the experimental group.
Trebaticka et al. [37]	*n* = 60 (7–18 years old). Inclusion criteria: diagnosis of MDD or MADD.	Group 1: Omega-3 FA-rich fish oil emulsion; Group 2: active comparator of Omega-6 FA-rich sunflower oil emulsion; Duration: 12 weeks.	CDI	The time-dependent treatment effect in the Om3 group was significant (F = 6.284, *df* = 6, *p* < 0.0001) with a significant decrease in CDI scores from baseline (*p* = 0.001 in week 2 to *p* < 0.0001 in week 12).
Gabbay et al. [38]	*n* = 51 (12–19 years old). Inclusion criteria: diagnosis of MDD.	Group 1: Omega-3 FA (max. dosages of 3.6 g/day); Group 2: placebo; Duration: 10 weeks.	CDRS-R, BDI-II	No superior efficacy of the experimental treatment to placebo in reducing the severity of symptoms of depression was observed.
Fristad et al. [36,39]	*n* = 72 (7–14 years old). Inclusion criteria: diagnosis of MDD, DD, or DD-NOS	Group 1: Omega-3; Group 2: PEP + placebo; Group 3: Omega-3 + PEP; Group 4: placebo; Duration: 12 weeks.	Primary outcome measure: CDRS-R; Secondary outcomes measures: BRIEF	Pre-post: The percentages of depressive symptom remission were: 77% for Omega-3 + PEP group; 61% for PEP + placebo group; 44% for the Omega-3 group; 56% for the placebo group. Follow up: Mood severity (CDRS-R: t = 2.13, *p* = 0.04, d = −0.035), executive functioning (BRIEF: t = −4.07, *p* = 0.000, d = 0.60), and the global one (CGAS: t = 3.89, *p* = 0.000, d = 0.63) continued to be significantly improved even if depressive symptoms (CDRS-R: t = 7.69, *p* = 0.002, d = −0.71) increased significantly from the end of the study to the follow-up.
Libuda et al. [40]	*n* = 113 (11.0–18.9 years old). Inclusion criteria: (1) diagnosis of hypovitaminosis D [25(OH)D ≤ 30 nmol/l]; (2) BDI-II score > 13	Group 1: TAU + oral Vitamin D_3_ (2640 IU/day); Group 2: TAU + placebo; Duration: 12 weeks.	Primary outcome measure: BDI-II; Secondary outcome measures: DISYPS-II, Serum total 25(OH)D	A greater increase in 25(OH)D levels in the experimental group emerged (treatment difference: +14 ng/mL; 95% CI 4.86–23.77; *p* = 0.003) while BDI-II scores did not differ significantly (+1.3; 95% CI −2.22 to 4.81; *p* = 0.466). A significant difference on the DISYPS scale by the patients’ parents was observed (−0.68; 95% CI −1.23 to −0.13; *p* = 0.016).

Legend: BDI-II = Beck Depression Inventory-II edition; BRIEF = Behavior Rating Inventory of Executive Functioning; CBCL = Child Behavior Check-List; CDI = Children’s Depression Inventory; CDRS-R = Children’s Depression Rating Scale-Revised; CGAS = Children’s Global Assessment Scale; CGI-I = Clinical Global Impression-Improvement; CGI-S = Clinical Global Impression-Severity; DD = Dysthymic Disorder; DD-NOS = Depressive Disorders-Not Otherwise Specified; DISYPS-II = Diagnostic System for Mental Disorders in Childhood and Adolescence, Self- and Parent Rating-II edition; FA = fatty acid; MADD = Mixed Anxiety Depressive Disorder; MDD = Major Depressive Disorder; PEP = Psycho-Educational Psychotherapy; TAU = Treatment As Usual; YMRS = Young Mania Rating Scale.

## Data Availability

The data presented in this study are available upon reasonable request from the corresponding author.
